# Cardiac Pain and Angina Pectoris

**Published:** 1897-11-27

**Authors:** S. H. Habershon

**Affiliations:** Assistant Physician and Pathologist to the Hospital


### Medical Progress and Hospital Clinics.

[*The Editor will be glad to receive offers of co-operation and contributions from members of the profession. All letters should be addressed to* The Editor, at the Office, 28 & 29, Southampton Street, Strand, London, W.C.]

### CARDIAC PAIN AND ANGINA PECTORIS.

A Clinical Lecture and Demonstration given at the Brompton Hospital for Consumption and Diseases of the Chest, by S. H. Habershon, M.D., F.R.C.P., Assistant Physician and Pathologist to the Hospital.

(*Continued from page* 133.)

**Vasomotor Angina of Peripheral Origin.**—In a second class of cases of so-called vasomotor angina the inducing cause of vasomotor spasm appears to be the condition of the blood.

The cases are not precisely of the same character. The main factor is peripheral. *Gouty patients* are extremely liable to a peripheral contraction of arterioles, which produces a high tension pulse, a distension of cardiac cavities, and cardiac pain. The early stages of such a condition are exceedingly remediable. I am inclined to think that the vasomotor affection is rather of peripheral than of central origin, though there are certain analogies between the condition and the uric acid neurosis that produces migraine.

Here, again, if the condition is allowed to continue, the high tension may induce an organic change in the cardiac muscle, and an angina pectoris which is directly referable to that organ.

In *senile cases,* the loss of elasticity in arterioles leads in many cases to a rise of pulse tension on slight provocation. In most cases no cardiac pain is produced, because the tendency is for the heart to hypertrophy in response to the peripheral resistance. It is difficult to dissociate the senile cases of angina pectoris from the cases of idiopathic disease, because peripheral resistance is only one of the many causes that produce arterial atheroma and sclerosis and an enfeebled, badly-nourished heart. Again, gouty atheroma is produced in old people by the long-continued effects of a hyper-acidity of blood upon the arterial and cardiac walls.

Some of these cases are alluded to by Powell under the name of "syncopal angina," and by Gairdner as "angina sine dolore." The characteristic is the tendency to sudden cardiac failure, especially from causes which call for increased cardiac action.

A condition allied to the senile peripheral resistance is to be found in *renal cases,* which frequently terminate with degeneration of the heart and cardiac pain and suffering.

Thus, the vasomotor cases gradually blend with the main group of cardiac origin, which I have now to describe. Remediable in the early stages, and so long as there is no cardiac lesion, but assuming the graver and more fatal type when the cardiac resistance is diminished by permanent vascular and muscular changes.

### B.—Cases of Cardiac Origin.

**Idiopathic Angina Pectoris.**—There are many points of great difficulty in the pathology and the true explanation of the symptoms produced in a typical case of angina pectoris. Such a case differs in its course and in its gravity from the vasomotory angina just described. The essential feature of true angina pectoris is that there is a cardiac spasm. It is agreed that the pain originates in a cardiac process, but opinions differ widely as to its pathology. In Heberden's original account the emphasis was laid upon the essentially spasmodic and paroxysmal character of the attack, and he classed the disease among the neuroses. Others, as Professor Gairdner tells us, have described it as a neuralgia of the heart or a hyperæsthesia of the cardiac plexuses. In neither case was sufficient stress laid upon the frequent association with organic disease of the cardiac muscle or arteries.

Dr. Lauder Brunton's brilliant researches upon the action of nitrite of amyl in an allied form of the disease have thrown much light upon the affection, though they do not elucidate all the difficulties. He found that the cardiac pain was accompanied by a pulse of such a character that it could only be explained by the supposition that there was a spasmodic constriction of the small arteries, and that a drug which had the effect of diminishing arterial tension would relieve the pain.

Another character which the typical disease shares with the slighter forms is that the pain usually extends to the left shoulder, arm, and fingers, and occasionally to the right also, thus showing that, through the cardiac plexuses, some of the spinal nerves are implicated, especially those in communication with the cervical and brachial plexuses.

In addition to these considerations, it is found that organic disease of the muscle of the heart, either fatty or fibroid degeneration, and frequently atheroma of the coronary arteries, with or without thrombosis, are present in fatal cases. None of these lesions are invariably present, but the frequent association of them with angina pectoris is sufficient ground for insisting, as Broadbent does, that degeneration, or imperfect nutrition of the cardiac tissues, is an inducing factor. Still, we must not forget that in many cases of death from other diseases extreme cardiac degeneration and coronary sclerosis are found without any history of pain during life. Brunton expresses the connection between the condition of pulse and the degenerate heart as follows: "Angina pectoris may be said to be due neither to high tension alone, nor weak heart alone, but to weakness of the heart in relation to the resistance it has to overcome, and it may be brought on either by weakening the heart, or increasing the resistance, or both together." The pulse in a typical case of angina pectoris is often small, thready, and of high tension—some, such as my colleague, Dr. Mitchell Bruce, have denied that the pulse is necessarily of great tension. This state of pulse is considered by Sir R. Douglas Powell to lend support to the view that the heart fails to close freely upon the blood and impel it into the arteries, and in extreme cases fails to expel it at all. In other cases, where the heart is vigorous and the peripheral resistance great, a slow and laboured pulse of high tension is found.

Angina pectoris in its typical form is a rare disease, though in its slighter forms it is common. I have one patient here who is under the care of Dr. Acland in the wards; and, though the distinction between the purely vasomotor type is not marked, I am inclined to regard her case as one of cardiac angina. She is 61 years of age. Her father and mother died suddenly at the ages of 71 and 81 respectively. She has had no illness until twelve months ago, when attacks of pain commenced. It is brought on by slight exertion, walking quickly, stooping, any excitement, and especially by exertion after a meal. The attacks of pain were at first more numerous than at present, as many as four or five occurring each day, and three or four in the night. The pain commences in the region of the heart, and rapidly extends over the chest to the left arm and hand, and to the right limb as far as the elbow. It is very severe, and she describes the sensation "as if everything was locked, and she would die." The breathing is laboured, and she sometimes coughs up frothy mucus, and frequently as the attack is passing off she vomits. The pain often lasts for an hour to an hour and a half. The attacks have been greatly relieved by small doses of iodide of potassium with the liquor trinitrinæ, and by tabloids of nitroglycerine. You will find a pulse of about 80, rather small, and of some tension. The heart is slightly enlarged and hypertrophied, the apex being in the fifth space, almost midway between the nipple line and the anterior axillary line. The second aortic sound is accentuated, and the first sound at the apex is soft and blowing. There is no doubt in this case that the physical signs point to a degenerative change in the cardiac muscle.

*(To be continued).*

